# Successful procedure with additional omentopexy to suture closure of gallbladder stump in laparoscopic subtotal cholecystectomy

**DOI:** 10.1111/ases.13007

**Published:** 2021-11-02

**Authors:** Hirotaka Kato, Hiroyuki Kinoshita, Masanori Kawaguchi, Hirofumi Yamazaki, Yoshifumi Sakata

**Affiliations:** ^1^ Department of Surgery Saiseikai Wakayama Hospital Wakayama City Japan; ^2^ Department of Gastroenterology Saiseikai Wakayama Hospital Wakayama City Japan

**Keywords:** bile leakage, laparoscopic subtotal cholecystectomy, omentopexy

## Abstract

Laparoscopic subtotal cholecystectomy, a bailout surgery for cholecystitis, can result in postoperative bile leakage, so surgical ingenuity is required. An 88‐year‐old woman had pain at the right hypochondrium. Abdominal computed tomography showed swelling of the gallbladder and thickness of the gallbladder wall, leading to diagnosis of mild acute cholecystitis. Percutaneous transhepatic gallbladder drainage was performed to alleviate cholecystitis because the patient was taking antiplatelet medicine. Laparoscopic cholecystectomy was then performed within 72 hours from the onset. The gallbladder was operatively found to be strongly fibrotic, so the procedure was switched to laparoscopic subtotal cystectomy, dissecting the gallbladder at the infundibulum‐cystic duct level. The gallbladder stump was closed with barbed suture and omentopexy was added due to fragility. There was no significant postoperative bile leakage. Additional omentopexy to stump closure in laparoscopic subtotal cholecystectomy was thought to be useful in prevention of postoperative bile leakage.

## 1 INTRODUCTION

Management for acute cholecystitis includes early performance of surgery when the surgical risk is lower, regardless of the severity or time from the onset. When surgical risk is higher, surgery should be performed as soon as cholecystitis has subsided by antibiotics treatment or drainage.^1^ Laparoscopic cholecystectomy, a standard procedure for acute cholecystitis, has high risk of intraoperative bile duct injury.^2^ Subtotal cholecystectomy can often be switched from cholecystectomy, although there is a 15.4% reported incidence of postoperative bile leakage.^3^ Laparoscopic subtotal cholecystectomy has comprised various treatments of the gallbladder stump to prevent postoperative bile leakage.^4^, ^5^ The most useful procedures have not yet been defined. We describe a procedure with additional omentopexy to stump closure, a brief and simple procedure that has not been reported, but could be a factor in preventing postoperative bile leakage.

## 2 CASE PRESENTATION

**FIGURE 1:**
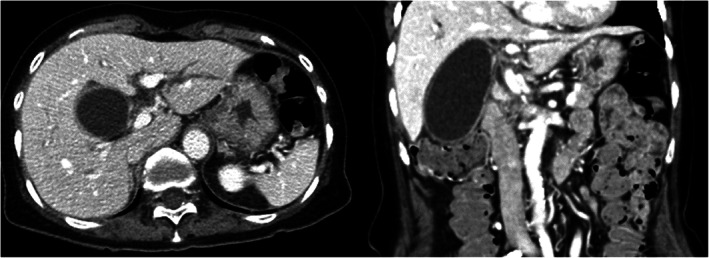
Abdominal computed tomography shows remarkable swelling of the gallbladder, thickness of the gallbladder wall and exclusion of the common hepatic duct, although there was no evidence of malignant tumors

An 88‐year‐old woman visited hospital with abdominal pain. She had a body temperature of 38.8°C and tenderness at the right hypochondrium. Blood examination showed white blood cell count 13 990/μL, C‐reactive protein 17.25 mg/dL, platelet count 26.7 × 10^4^/μL, prothrombin time (PT) 84.8%, PT – international normalization ratio 1.05, activated partial thromboplastin time 34.2 seconds, aspartate aminotransferase 18 IU/L, alanine aminotransferase 13 IU/L, alkaline phosphatase 80 IU/L, γ‐glutamyl transpeptidase 17 IU/L, total bilirubin (T‐Bil) 1.9 mg/dL and direct bilirubin (D‐Bil) 0.2 mg/dL. Abdominal computed tomography (CT) showed remarkable swelling of the gallbladder, thickness of the gallbladder wall and exclusion of the common hepatic duct, but no malignant tumors (Figure 1). Mild acute cholecystitis was diagnosed according to guidelines. The patient was taking antiplatelet medicine (cilostazol) for cerebral infarction, and the time from onset was just within 24 hours. Treatment strategy was therefore to stop the antiplatelet medicine and to perform percutaneous transhepatic gallbladder drainage (PTGBD) to relieve the swelling due to cholecystitis. Laparoscopic cholecystectomy was then planned for within 72 hours of the onset of cholecystitis.

### 2.1 Operative finding

**FIGURE 2:**
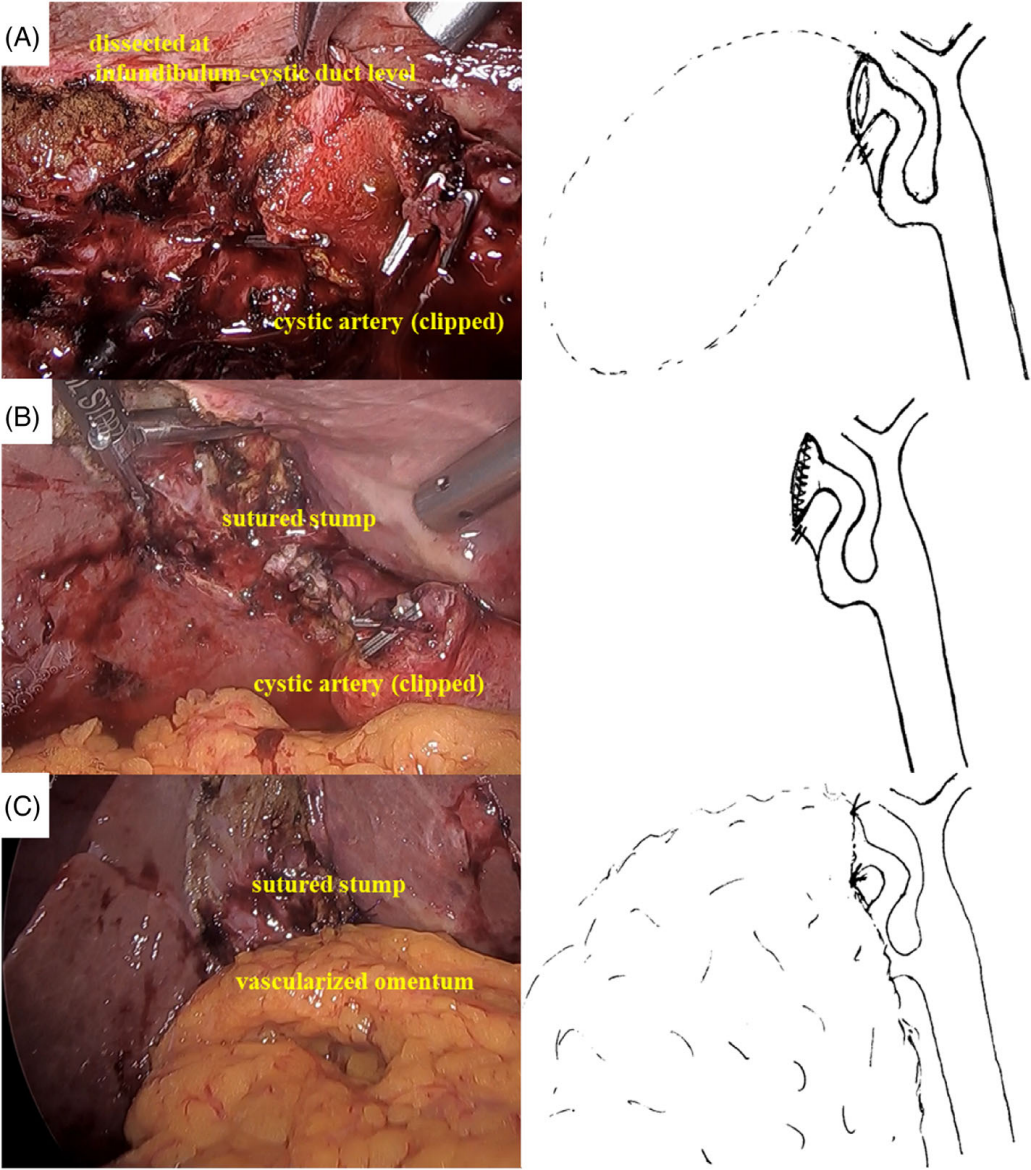
(A) The gallbladder was dissected at the infundibulum‐cystic duct level. (B) The gallbladder stump was sutured continuously with 3/0 V‐Loc, without ligating the orifice of cystic duct and without performance of mucoclasis of the remnant gallbladder. (C) Vascularized omentum was mobilized and used to cover the stump by two stitches on the cranial and caudal side of the stump with 3/0 vicryl

Laparoscopic cholecystectomy was begun, and the omentum around the gallbladder was found to have adhesion to the abdominal wall. We towed the gallbladder, which had poor mobility around the gallbladder neck. The cystic artery could be clipped and dissected. The cystic duct was strongly fibrotic and scarred, so the gallbladder was dissected at the infundibulum‐cystic duct level (Figure 2A). The residual gallbladder was fragile, so the gallbladder stump was sutured continuously with 3/0 V‐Loc (Covidien, Medtronic, Mansfield, MA, USA) (Figure 2B), without ligation of the orifice of the cystic duct or performance of mucoclasis of the remnant gallbladder. Stump closure only was thought to be insufficient to prevent postoperative bile leakage, so the vascularized omentum was mobilized and used to cover the stump with two stitches at the cranial and caudal sides of the stump with 3/0 vicryl (Figure 2C). A drainage tube was placed at the stump, and the operation was completed after confirmation of no significant bile leakage. Pathological diagnosis was acute cholecystitis without malignant tumor.

Drain discharge on postoperative day (POD) 1 showed that T‐Bil was 0.78 mg/dL and D‐Bil was 0.15 mg/dL, suggesting no significant bile leakage. The drain was removed on POD 2. The patient had a favorable postoperative course and was discharged on POD 5.

## 3 DISCUSSION

Laparoscopic cholecystectomy for acute cholecystitis requires critical view of safety (CVS), used to fully visualize the cystic duct and the cystic artery after peeling off the Calot triangle to prevent bile duct injury. The subserosal (SS) layer of the gallbladder sometimes progresses to fibrosis and scarring under cholecystitis, and the boundary between SS‐inner layer and SS‐outer layer becomes unclear,^6^ making CVS difficult.^2^ Bailout surgery should thus be considered in such cases. Subtotal cholecystectomy, a kind of bailout surgery particularly from incomplete cholecystectomy, has several variations including dissection that not only concern the cystic duct, but also the junction between the gallbladder or infundibulum and the cystic duct. Meanwhile, there is a reported frequency of 0.7% of incidental diagnosis of gallbladder carcinoma during laparoscopic cholecystectomy.^7^ The contents of the gallbladder leak into the abdominal cavity during surgery, resulting in dissemination of tumor cells under the presence of malignant tumors. Evaluation of malignant tumors with preoperative CT is therefore important.

In the present case, abdominal CT showed no significant malignant tumor in the gallbladder. The patient was taking antiplatelet medicine and the gallbladder was remarkably swollen, which made it difficult to perform emergent surgery owing to intraoperative bleeding and surgical operability. The patient moreover visited hospital within 24 hours from onset of cholecystitis. PTGBD was therefore planned prior to the surgery and the operation was to be performed within 72 hours from onset. Regarding intraoperative findings, the procedure was switched to subtotal cholecystectomy at the infundibulum‐cystic duct level due to the difficulty of peeling off the Calot's triangle and in preparing CVS.

Laparoscopic subtotal cholecystectomy also has a higher incidence of postoperative bile leakage,^8^ because edematous cystic ducts or gallbladder stumps due to cholecystitis are at risk of bile leakage when the edematous tissue is reduced or when the gallbladder stump loses strength after surgery. Appropriate treatment of the cystic duct or gallbladder stump can reduce the risk of postoperative bile leakage. Fujiwara et al^9^ reported the usefulness of barbed sutures for the stump. A continuous suture with barbed suture can adjust not only the suture pitch and bite, but also the tension according to the thickness, strength and flexibility of the edematous tissue. Matsui et al^5^ reported the use of free omentum tissue being plugged into the orifice, and then closure with moderate tension with absorbable suture clip. The present procedure is less likely to become necrotic than with an omentum plug. In the present procedure, a more sufficient field of view at the fixed part with the omentum can be secured not to fall off when covering the vascularized omentum after closure of the stump. As for the advantages of additional omentopexy, Lale et al^10^ reported that staple line reinforcement with omentopexy is a promising method for the prevention of postoperative leakage in laparoscopic sleeve gastrectomy.

In the present case, the omentum around the gallbladder was fragile due to cholecystitis. However, the gallbladder stump could be successfully covered with a normal and thick vascularized omentum by mobilization. The present simple surgical procedure has not been previously reported in cholecystectomy. It could be a factor in preventing postoperative bile leakage, even under acute cholecystitis.

In conclusion, stump closure in subtotal cholecystectomy with additional omentopexy was thought to be successful in prevention of postoperative bile leakage. Larger studies of the association between this surgical procedure with postoperative bile leakage are required.

## CONFLICT OF INTEREST

All authors declare they have no competing interests.

## AUTHOR CONTRIBUTIONS

Hirotaka Kato collected data and wrote the manuscript. Hiroyuki Kinoshita, Masanori Kawaguchi, Hirofumi Yamazaki and Yoshifumi Sakata read and helped write manuscript. All authors have read and approved the final manuscript.

## CONSENT FOR PUBLICATION

Written informed consent for publication of the present article and clinical data was obtained from the patient.

## Data Availability

The data that support the findings of this study are available from the corresponding author upon reasonable request.
